# Recent Developments in the Application of Filamentous Fungus *Aspergillus oryzae* in Ruminant Feed

**DOI:** 10.3390/ani14162427

**Published:** 2024-08-21

**Authors:** Clarisse Uwineza, Milad Parchami, Mohammadali Bouzarjomehr, Mohammad J. Taherzadeh, Amir Mahboubi

**Affiliations:** Swedish Centre for Resource Recovery, University of Borås, 50190 Borås, Sweden; clarisse.uwineza@hb.se (C.U.);

**Keywords:** edible filamentous fungi, *Aspergillus oryzae*, ruminant feed, ruminal fermentation, sustainable feed

## Abstract

**Simple Summary:**

Ruminant farming, which includes animals like cows and sheep, is resource-intensive and this is becoming more of an issue as the human population grows. People are also worried about the use of antibiotics in animal feed. Scientists are looking for new, sustainable ways to feed these animals to improve their health and production without antibiotics. One promising solution is using edible fungi, like *Aspergillus oryzae*. This fungus can provide important nutrients, such as proteins, which are usually obtained from plants or animals. By adding these fungi to animal feed, it can reduce the need for conventional protein sources and antibiotics, enhancing the quality of the feed and the well-being of the animals. This review paper explores the effects of including an *Aspergillus oryzae* fungal biomass in ruminant feed and explains how this fungus can be grown on organic residues. In summary, using *Aspergillus oryzae* in animal feed provides essential nutrients that can support animal health and productivity, all of which are valuable to society.

**Abstract:**

The resource-intensive nature of the ruminant farming sector, which has been exacerbated by population growth and increasing pressure to reduce feed antibiotics and growth promoters, has sparked interest in looking for sustainable alternative feed sources to enhance ruminant production efficiency. Edible filamentous fungi, rich in macronutrients like proteins, offer promise in reducing the reliance on conventional protein sources and antimicrobials to improve feed quality and animal performance. The inclusion of single-cell proteins, particularly filamentous fungi, in ruminant feed has long been of scientific and industrial interest. This review focuses on the potential application of the extensively studied *Aspergillus oryzae* and its fermentation extracts in ruminant nutrition. It provides an overview of conventional ruminant feed ingredients, supplements, and efficiency. Additionally, this review analyzes the re-utilization of organic residues for *A. oryzae* cultivation and examines the effects of adding fungal extracts to ruminant feed on ruminal digestibility and animal performance, all within a circular bioeconomy framework.

## 1. Introduction

The consequences of population growth in recent decades have been accelerated urbanization and income growth, causing a continuous change in preferred diet and an increase in global food demand. This is reflected in every part of the food supply chain, including the demand for livestock products [1,2]. Livestock products (e.g., meat, dairy products, eggs, fish, etc.) play a crucial role in achieving a balanced diet by providing about 33% of the global protein requirement and 17% of the human calorie intake [3,4]. Current global estimations assert that, to meet the growing population’s food demands by 2050, food production would have to increase by 50%, of which 70% will come from animal products [2,5]. In this regard, there is a general consensus that increased global food and animal feed protein demands will substantially increase the need for commodities, especially cereals and meat [6]. The Food and Agriculture Organization (FAO) estimates that the world cereal production for both food and animal feed will need to increase by up to 940 million tons annually from the current 2.1 billion and the global meat production will need to rise from about 200 million tons annually to nearly 455 million tons by 2050 [7]. The increasing future demand for animal-based protein will impose significant demands on feed provision resources. For instance, animal feed protein sources count for 70 million tons from food grade protein annually for livestock production [8].

The drive to produce edible plant-based protein to meet the demand for animal feed is associated with increased water and arable land use, leading to land degradation, deforestation, water scarcity, and other environmental concerns. This may also, in extreme cases, give rise to ethical issues, such as food versus feed, and a rise in food and livestock prices [9]. The increase in cereal prices may be partly due to the fact that about 900 million tons of cereals are currently used in animal feed, a number that is projected to reach 1.1 billion tons by around 2050. It should be noted that 85% of produced soybean meal is used for animal feed that can also be of value to the human diet [10]. Soybeans are an important crop globally. In the European Union, most protein meals in livestock diets come from imported soybean, primarily from Brazil and the United States. In 2019, 64% of Europe’s soybean demand was provided by Brazil and the USA [11]. Over the next decade, soybean production is projected to increase annually by 0.7% in Brazil and 0.5% in the USA. In addition, agriculture land use in Brazil is anticipated to expand by nearly 4 million hectares, with 31% allocated to soybeans [12]. This steady increase has caused widespread deforestation, biodiversity loss, and increased carbon emissions. For example, from 2009 to 2017, soybean cultivation accounted for 29% of deforestation in the amazon biome, and, in 2016, land use changes in the Cerrado region alone released about 248 million of CO_2_ emissions [13]. Furthermore, 12% of greenhouse gas emissions from the international trade of livestock feed in Europe were attributed to Brazilian soybeans [11].

In response to the above-mentioned concerns, the FAO has highlighted the integration of organic residuals to produce sustainable alternative protein sources for animal feed with the potential to replace the conventional protein sources (e.g., soybean meal, grains, and cereals) currently used in animal feeding [14]. This means transforming residual materials, including crop residuals and the by-products of food industries, into alternative protein-rich feedstuff, such as insects and single-cell protein (SCP) [15]. This way of improving sustainable livestock feed production will largely bring about environmental changes, particularly in the scope of sustainable arable land use, and improve the waste management policies that convert low-quality materials to value-added resources, contributing positively to achieving a circular economy [16]. SCPs from specific strains of bacteria, yeast, filamentous fungi, and algae are considered to be safe for humans, since they have been used in production in the food and beverages industries for decades.

In recent decades, SCPs have gained much attention as a potential alternative protein substitute for human food and animal feed supplementation [17]. In addition to their high protein content, ranging between 40 and 82% on a dry matter basis, SCPs can deliver a wide range of valuable vitamins, carbohydrates, lipids, minerals, and essential amino acids [17,18]. SCPs can be produced by bacteria (*Cellulomonsa*, *Alcaligenes*, etc.), algae (*Spirulina*, *Cholorella*, etc.), yeast (*Saccharomyces*, *Candida*, etc.), and fungi (*Rhizopus*, *Fusarium*, *Aspergillus*, etc.) [18,19]. The choice of the microorganism used for the production of SCPs depends on factors such as the growth media type and composition, production process, and final nutritional requirements in the feed/food [19]. Bacteria can utilize a wide range of carbon sources and nutrients to produce a high protein biomass (usually 40–80%) that is rich in amino acids such as cysteine and methionine but deficient in glycine. However, the main disadvantages of bacteria are the high nucleic acid content, difficulty in harvesting and concentration due to small size and low cell density, issues related to palatability, and the high demands in various applications that increase the cost of development [17,18]. Algal microbial biomass can contain up to 70% protein, is mainly rich in cysteine and methionine, and is easy and fast to cultivate. In addition to the possibility of accumulating heavy metals from growth media, algal microbial SCP production processes are minimal due to production costs and demands for CO_2_, sunlight, and warm temperatures [17,18,19]. Yeast cells are also a good source of SCP production and can contain up to 65% crude protein. They are low in methionine and nucleic acid and high in lysine compared to microbial bacterial biomass. Filamentous fungi are beneficial due to their versatility in the production of valuable compounds such as organic acids, enzymes and SCPs, using a wide range of organic agricultural products and industrial waste. The produced fungal biomass can contain up to 60% crude protein. They are rich in lysine and threonine but deficient in the sulfur-containing amino acids cysteine and methionine. Despite their initial growth rate, they are easier to harvest compared to bacteria, yeast, and algae [17,18]. In addition, yeast (e.g., *Saccharomyces cerevisiae*, *Candida utilis*) and edible filamentous fungi (e.g., *Aspergillus oryzae*, *Aspergillus niger*, *Fusarium venenatum*) have leverage over bacteria and algae as they have long been used in the food, beverage, and medicine industries.

*Aspergillus oryzae* (*A. oryzae*) is one of the most well-studied and commercially applied edible filamentous fungi that inherited its fame from application in producing shoyu, sake, and miso [20]. *A. oryzae* can utilize a wide range of organic-rich residuals from agriculture and food industries to produce high-value fungal biomass, making it a good candidate as an alternative protein and nutrient source in animal feed. The medium constituents determine the nutritional value of *A. oryzae* biomass, including protein, minerals, and polyunsaturated fatty acids. For instance, the filamentous fungal biomass produced from substrates rich in carbohydrates but poor in nitrogen sources has been reported to contain more lipids and less protein [21]. Regarding its potential application in animal feed, *A. oryzae* is also considered a prebiotic additive mainly used as a direct-fed microbial; a fermentation extract of *A. oryzae* known as Amaferm^®^ (BioZyme Inc., St. Joseph, MO, USA) and a combination with *A. niger* known as Fibrase^TM^ or Fibrase^TM^ farm when yeast is added (Balchem Animal Nutrition & Health, NY, USA) are available on market and widely studied and used as a microbial feed additive to improve ruminant performance [22,23]. The addition of *A. oryzae* to ruminants’ diets during in vivo and in vitro assessment improved feed intake, increased the number of rumen cellulolytic bacteria by increasing the rate of fiber and dry matter digestion [24], enhanced the ruminal digestive tract, increased volatile fatty acid (VFA) concentration as a metabolizable energy supply, and improved milk production efficiency [23,25,26]. In addition, during fungal fermentation, enzymes and other metabolites are produced and contribute to the neutralization or degradation of mycotoxins and antinutritional factors like tannins, and phytic acid makes the feed more digestible, nutritious, and safe for animal consumption [27].

Although fungal SCP is an excellent sustainable protein source, economic production feasibility remains challenging. Application of SCPs in animal feed requires developments in production technology and a profound exploration of their digestibility and effect on animal physiology [18,19]. The production costs for SCPs are not comparable to that of conventional feedstuff such as soy, rapeseed, and corn. As SCPs mainly have a higher protein content and better amino acid profile compared to their conventional counterparts, a decrease in production costs and cultivation rate enhancement could be a game changer in animal feeding.

This review provides an interesting insight into recent findings regarding the application of filamentous fungi and its fermentation extracts and metabolites in ruminant feeding. It offers a complete overview of ruminant feed and additive composition, fungal biomass cultivation requirements, and potential organic substrates and their characteristics. A great focus has been given to the application of single-cell proteins, specifically *A. oryzae*, in ruminant feed, its digestibility in ruminants’ digestive tracts, and direct and indirect effects of SCPs on ruminant products such as growth, rumen microbiota, milk yield, composition, etc. Finally, the review thoroughly discusses the perspective on the application of fungal SCPs in ruminant feeding within a circular bioeconomy concept.

## 2. Ruminant Feed Ingredients, Supplements, and Their Efficiency

### 2.1. Feed Ingredients and Supplements

**Figure 1:**
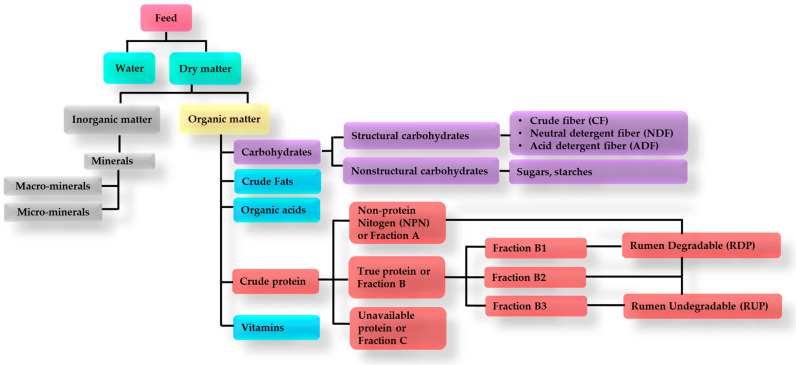
Comprehensive nutritional composition of ruminant feed.

Feed provision has some of the highest expenses in the livestock sector. The nutrients in feedstuffs influence animals’ maintenance, growth, and reproduction. However, not all elements of feedstuff are of equal nutritional value; some are indigestible, not absorbable, or even toxic to the body. Essential nutrients are carbohydrates, fats, proteins, minerals, and vitamins [28] as illustrated in Figure 1.

Feed classification involves categorizing feed into groups with similar nutritional values and applications. This categorization is useful for predicting the quality and potential of novel feed or in choosing alternative options for conventional ingredients that are no longer easily accessible. Animal feed can be classified in several ways. The International Network of Feed Information Centers (INFIC) categorizes feeds into eight classes [29]: dry forages and roughages (have more than 18% crude fiber in dry matter), pasture, forages, and range plants that are not cut and processed, silages, energy feeds (containing less than 18% crude fiber and less than 20% protein), protein supplements (containing less than 18% crude fiber and 20% or more protein), mineral supplements, vitamin supplements, and additives (non-nutritive materials incorporated into feeds). In addition to National Research Council (NRC) publications on feeding standards, many other references use this classification [30].

Plant-derived materials make up about 70–90% of the components used in formulating industrial animal feed. The principal ingredients originate from cereals and their by-products from diverse industries, followed by protein-rich plant materials such as legumes. Animal by-products, including meat and bone meal and fat, for instance, tallow or lard, could be included to a lesser extent. Also, additives such as vitamins, trace elements, synthetic amino acids, growth promoters, and antibiotics complete the diet [31].

The energy provided by the feed invigorates all body functions that enable the animal to perform multiple operations, including growth, body maintenance, and production. Metabolizable energy is the energy available from the feed after subtracting the energy lost in feces, urine, and gas. The metabolizable energy depends on the quality and digestibility of the feed [32]. Carbohydrates and fat are the primary sources of energy in the diet. Carbohydrates are particularly important for ruminants, typically 60 to 70% of their total diet. They provide energy for rumen microbes and different parts of the animal’s body; besides that, specific carbohydrates contribute to the health of the gastrointestinal tract [33]. Carbohydrates are generally categorized as either structural or non-structural. Non-structural carbohydrates are more digestible and include sugars and starch [33,34]. The most common energy supplement for cows fed in pasture-based systems is cereal grains. Generally, grains have a high content of rumen-degradable protein and a high content of non-structural carbohydrates. The rumen protein synthesis can be enhanced by fermentable protein and improved protein–energy balance provided by starch-based concentrates [35,36].

Another source of energy in the diet is fat. Fat is generally applied as a non-exclusive term to define compounds with great content of fatty acids (FAs) containing triglycerides, phospholipids, non-esterified FAs, and long-chain FA salts. Fat can be fed to ruminants in various forms, such as oilseeds, animal–vegetable fat mixtures, and granular fats. Fat supplementation increases the energy mass of the diet and enhances fat-soluble nutrient intake [34]. Metabolizable energy, obtained from energy sources (carbohydrates and fats), is primarily used for body maintenance and to a lesser extent for growth, fattening, and production [37].

Recently, there has been an increasing demand for supplementation of cereal grains due to the increased genetic merit of dairy cows. Nevertheless, feeding immense portions of grains can negatively impact ruminal fiber digestibility, reduce ruminal pH, and alter the acetate/propionate ratio, leading to a higher incidence of ruminal acidosis [38]. To remediate these issues and to boost energy intake, interest has risen in supplementing dairy cows’ diets with fat [39]. Fat supplementation can enhance palatability and feed efficiency and assist in heat stress reduction [40]. Specific FAs such as conjugated linoleic acid may affect nutrient partitioning [41] and reduce dust generated during feed processing [42]. In addition, increasing the intake of feeds rich in non-structural carbohydrates can promote propionate production contributing to the increase in VFA production and supplying more energy to host animals [43].

Proteins provide the structural material for body cells and tissues (blood, muscles, and skin). Dietary protein is accounted as crude protein containing protein and non-protein nitrogen. Protein is divided into rumen-undegradable protein (RUP) and rumen-degradable protein (RDP). RUP is formed of the protein fraction that passes through the rumen without biodegradation, while RDP is the protein portion of the feed providing nitrogen to rumen microorganisms to thrive [33]. In addition, components such as urea and ammonium salts are considered non-protein fractions of crude protein that supply readily available nitrogen to rumen microorganisms [33,37]. Furthermore, the Cornell Net Carbohydrate and Protein System (CNCPS) classifies crude protein into three fractions: non-protein nitrogen (NPN or A), true protein (B), and unavailable nitrogen (bound true protein) (C). True protein (B) is further divided into three subfractions (B1, B2, and B3) based on their ruminal degradation rate. Fractions A and B1 rapidly dissolve and degrade in the rumen, B2 degrades partially, and B3 degrades extra slowly in the rumen. Fraction C consists of proteins resistant to microbial and enzyme degradation, such as those bound with lignin, tannin–protein complexes, and Maillard products, and they do not provide postruminal amino acids [44].

A complex of micro- and macrominerals is essential in a ruminant’s diet. Minerals are necessary for the body to function properly and maintain production and reproduction [37]. A balanced intake of minerals such as calcium, phosphorus, chloride, sodium, and potassium and trace elements like copper, zinc, selenium, chromium, and iodide is crucial for dairy cow reproduction. These minerals’ various physiological functions include bone development, muscle contraction, reproduction, and lactation. For instance, calcium and phosphorus are critical for skeletal and muscle contractibility, while sodium and potassium are vital for maintaining osmotic balance, most importantly in alleviating heat stress in ruminants [45]. Macrominerals including calcium, magnesium, phosphorus, potassium, sodium, and chlorine are inorganic elements required in gram quantities or as a percentage of the dry matter in the diet. On the other hand, microminerals are needed in milligram or microgram amounts such as chromium, copper, iron, selenium, and zinc [46]. According to the Council [33], the quantities of essential minerals required in ruminants’ diets differ based on the animal’s productivity level and physiological state. For instance, for diets of young calves, calcium concentrations of 1%, 0.70%, 0.60%, and 0.95% are recommended in milk replacer, starter feed, grower feed, and whole milk, respectively.

Vitamins are required by the body in small quantities to help ruminants utilize other nutrients effectively. They play vital roles in various body processes such as cooperation in different metabolic pathways, maintaining healthy protective tissues, enhancing appetite, bone formation, growth, and preventing anemia [33,46]. Vitamins are divided into fat-soluble and water-soluble groups. Because simple rations (mainly corn and soybean meal) often lack vitamins such as A, K, and D, supplements must be added to the diet.

### 2.2. Feed Efficiency

One approach to increasing feed utilization efficiency is the addition of catalytic amounts of strategic nutrients, such as trace minerals, vitamins, amino acids, and other bioactive compounds to the basic ration. Adding these nutrients increases the activity of the native microbial population in the rumen, leading to improved rumen fermentation and overall performance of the animals. The deficiencies in essential nutrients in feed are generally compensated by supplements [47]. Some roughage, such as grass, is of poor quality and incompetent for sustaining high levels of production. In certain regions where pasture or grasses are the only accessible feed sources for ruminants, deficiencies in trace elements, vitamins, proteins, and other nutrients are common. The nutrient requirements of cattle depend on factors like weight, production rate, genetics, and environmental conditions. Nutritionists theoretically use the nutrient content of the ration to identify deficiencies and design supplements to provide sufficient minerals, vitamins, protein, and energy. A congruous, high-quality feed is required at all times for efficient production; merely filling the stomach is not enough. Both intensive and extensive animal production systems can benefit from additives that have been developed through biotechnology. Vitamins, trace minerals, growth inducers, bypass protein supplements [48], rumen-protected amino acids, probiotics [49], and disease-prevention agents are just a few of the additives used in the animal production industry. 

Furthermore, increasing feed efficiency also involves enhancing the protein fraction in feed with respect to its quality and rate of digestibility. Rumination is one of the parameters that significantly contributes to feed efficiency in ruminants. It is a phenomenon that involves chewing the cud or rechewing the content previously ingested in the rumen, leading to better nutrient absorption and overall digestive health. High-fiber feeds such as hay, silage, and pasture increase rumination time which is essential for stimulating chewing and saliva production, crucial for buffering rumen pH and promoting healthy rumen function [50]. Restricting dry feed availability could be used to extend rumination time and change daily pH patterns, thereby enhancing the digestive efficiency of lactating cows [51].

### 2.3. Protein Supplementation in Ruminant Feed

The fundamental purpose of including protein in the ration is to optimize the conversion efficiency of dietary nitrogen into animal products such as milk and meat. Two approaches must be employed to fine-tune protein nutrition in ruminant feeding. Firstly, the right type and quantity of RDP should be provided to meet but not exceed the nitrogen requirements of ruminal microbes. Second, it is essential to feed RUP with a balanced amino acid profile (AA) profile that complements the ruminal synthesized microbial protein. When combined with microbial protein, these proteins provide the preferred protein concentration based on the absorption ratio of energy:protein [52]. However, pure amino acids and protein supplements are easily degraded by rumen microbes, making them inadequate on their own. The development of a rumen protection system for amino acids and protein supplements can be a solution. Over the past few decades, various approaches for protecting amino acids and protein have been investigated. For protecting amino acids, strategies such as using polymers and natural substances for coating, metal chelation for complexation, and N-modification and iso-peptide bonds for chemical modification have been explored [53,54].

Multiple techniques have been studied to decrease the rate and extent of protein degradation in the rumen [55,56]. Since 1954, when Chalmers and Cuthbertson [57] first applied heat treatment, it has become one of the most common processes for protecting proteins from rumen degradation. The expeller processing of oilseeds (in disparity with solvent extraction), extrusion, and roasting have been incorporated into the heating process for shielding proteins [58,59]. Several chemicals, including tannins, aldehydes, alcohols, sodium, different cations, and reducing sugar, have lowered ruminal protein degradation. These compounds act by creating pH-dependent modifications that lessen protein degradability. However, with proper processing, the degradation of proteins can start in the acidic environment of the abomasum and proximal duodenum [60].

### 2.4. Microbial Supplementation in Ruminant Feed

A broad spectrum of chemical feed additives has been used in ruminant nutrition to modify the microbial community and fermentation characteristics in the rumen and intestine of ruminants, including antibiotics, ionophores, methane inhibitors, and defaunating agents [61]. As a result of public awareness of issues such as bacterial resistance to antibiotics due to increased utilization of these chemicals in the food chain, their use as feed additives has been restricted by a growing number of jurisdictions [62]. Probiotics containing organisms capable of living in the rumen environment have become a suitable alternative to chemical feed additives in response to consumer demands [63]. As a result, the US Food and Drug Administration has demanded feed manufacturers employ the terminology “direct-fed microbial (DFM)” instead of probiotics [64], while Yoon and Stern [65] have limited it to “a source of inherently live microorganisms”. Specific and non-specific yeast, fungi, and bacteria can all be included in the definition of DFMs [66]. Some parameters, such as the amount of DFMs, feeding frequency, and types of DFMs, determine the DFM mode of action [67]. It is believed that the main role of DFMs in the rumen is through lactic acid-producing (LAB) or lactic-acid-utilizing bacteria (LUB). LAB boost the growth of LUB and other microorganisms in the rumen that are adapted to the existence of lactic acid, which helps prevent ruminal acidosis and stabilizes ruminal pH [65,68]. Lactate-utilizing bacteria, such as *Megasphaera elsdenii*, prevent pH falls driven by lactate accumulation [69].

The DFM function in the gastrointestinal tract is elucidated in the literature [65]. Hydrophobic interactions enable DFMs to interfere with or prevent the attachment of pathogens like *Escherichia coli* on the intestinal mucosa and bind to enterocyte receptors or produce enterotoxins that provoke diarrhea [70]. Frizzo, Soto [71] claimed that LAB have protection properties against salmonella in animals by attaching to the intestinal tract. Apart from LAB’s function of producing lactate and acetate as metabolic end-products, LAB’s role is critical in impairing cellular functions by infiltrating microbial cells [72]. Fungal DFMs are widely used in ruminants to improve performance and regulate rumen fermentation [73]. The most employed fungal species as DFMs are *S. cerevisiae* and *A. oryzae* [74]. Yeast may act as a rumen pH stabilizer by mediating sharp decreases in the rumen pH [74]. By providing dicarboxylic acids (e.g., malic acid) and other growth factors such as amino acids, peptides, vitamins, and organic acids, fungi such as *A. oryzae* can enhance lactate utilization by ruminal organisms such as *Selenomonas ruminantium* [75]. In addition, supplementing with *A. oryzae* fermentation extract improves fiber, crude protein, and DM digestibility, enhances rumen microorganism populations, and increases volatile fatty acid production and hence stabilizes the pH of the rumen [76,77].

## 3. Filamentous Fungi Cultivation, Composition, and Characteristics

### 3.1. Filamentous Fungi Cultivation

For centuries, filamentous fungi have been exploited in various biotechnological applications, including food and beverage production [78]. Fungal biomass is an excellent source of proteins, amino acids, vitamins, lipids, and cell wall materials, including mannoproteins, β-glucans, chitin, and biodegradable fibrous polysaccharides. Filamentous fungi (*Aspergillus*, *Fusarium*, *Rhizopus*, and *Mucor*) and yeast (*Saccharomyces*, *Candida*, *Rhodotorula*, and *Pichia*) have been extensively used in fermentation processes to produce fuels, food, and animal nutrients [78,79]. However, the nutrient composition of filamentous fungi and yeast can vary based on factors such as the substrates used for fungal cultivation, cultivation conditions, and the postfermentation extraction process. Fungal cultivation is generally conducted in submerged or solid-state fermentation. Parameters influencing the cultivation outcome are medium composition, mode of fermentation (submerged or solid state), strain type, mass transfer (agitation conditions and medium viscosity), temperature, pH, aeration, harvesting, and extractions [79,80]. The interaction of the parameters defines the productivity of fungal cultivation [80,81]. Besides the fungal strain, the medium composition, including the type of carbon and nitrogen source, phosphate content, carbon-to-nitrogen ratio, insoluble particles, and inhibitory compounds, directly affect fungal growth, substrate bioconversion rate, productivity, and fungal biomass composition (protein, lipids, fibers, minerals, and vitamins). It should be noted that enhanced fungal bioconversion requires optimization of these factors [81].

Generally, most fungi are fast simple sugar (glucose, fructose, etc.) assimilators. Synthetic media containing sugar-based substrates (glucose, maltose, and starch) supplemented with nutrients such as ammonium and urea are commonly used to cultivate filamentous fungal biomass. Glucose, in particular, has been extensively researched and widely applied as a carbon source for the growth of filamentous fungi such as *Aspergillus oryzae*, *Aspergillus niger*, *Neurospora intermedia*, *Rhizopus oligosporus*, and *Rhizopus oryzae* and yeast such as *S. cerevisiae*. Filamentous fungi are versatile microorganisms with efficient enzymatic machinery that can secrete hydrolytic enzymes such as cellulase, amylase, proteases, and lipase, allowing them to metabolize complex substrates. Such characteristics confer filamentous fungi’s unique ability to break down carbohydrates, proteins, and lipids from various substrates to use them as nutrient sources. Nitrogen- and phosphorus-bearing compounds are key factors that influence fungal cell growth, carbon source consumption, and the metabolism of carbohydrates and nucleic acids. These elements play a critical role in the production of secondary compounds, thereby affecting protein, lipid content, and overall composition of the final fungal biomass.

The pH of the media is a crucial growth-defining parameter. For instance, *A. oryzae* can germinate at a pH range of 2 to 8, with optimal growth occurring between pH 5 and 7 [82]. The optimal pH of growth for *A. oryzae* in a volatile-fatty-acid-rich effluent is between 6 and 7 [83], while it could grow well at pH between 5 and 6 in glucose [84] and other effluents such as vinasse [85], spent liquor [86], wastewater [87], and potato protein liquor [88].

### 3.2. Aspergillus Oryzae Cultivation Potentials

*A. oryzae*, the most studied edible filamentous fungi for inclusion in ruminant feed, possesses a powerful enzymatic machinery enabling it to secrete different enzymes such as alpha amylase, beta amylase, proteases, and cellulase and is used to break down and consume a variety of substrates such as complex waste streams [89,90]. Therefore, *A. oryzae* has proven to be cultivated and grown using different substrates on both in solid-state (SS) and submerged (SM) cultivation modes [91] to produce fungal biomass with a high protein content [92]. As presented in Table 1, the potential of *A. oryzae* in producing protein-rich biomass using different substrates has been investigated extensively.

#### 3.2.1. Ethanol Industry Residues for *A. oryzae* Cultivation

For example, first-generation ethanol production plants generate different organic wastes and by-products, such as stillage (whole or thin stillage), and vinasse which can be used for feed-grade single-cell production. In this context, Bátori, Ferreira [104] reported that whole stillage, a by-product of the dry-mill ethanol process, can be used to produce *A. oryzae* under optimal conditions of 35 °C and pH 5. In addition, Ferreira, Lennartsson [90] reported that thin stillage, which mainly contains the soluble fraction of whole stillage, is a promising substrate for the production of 19 g/L of *A. oryzae* fungal biomass containing 48% protein with a high reduction in final glycerol content in the fermentation broth. Karimi, Mahboobi Soofiani [85] used vinasse as an organic-rich residue from distillation of ethanol fermentation broth to produce *A. oryzae* fungal biomass. They reported that under optimal conditions (35 °C and pH 6), a biomass with 44.7% protein content can be achieved, while a 95% (*v*/*v*) diluted vinasse provides the highest fungal growth rate.

#### 3.2.2. Pulp and Paper Industry Residues for *A. oryzae* Cultivation

Industrial wastes can sometimes serve as an organic basis for single-cell production. In this regard, Asadollahzadeh, Ghasemian [86] observed that black liquor, a by-product of the pulp and paper industry, can be used as a promising substrate for cultivation of fungal biomass. They reported that among different filamentous fungal strains, *A. oryzae* achieved the highest biomass production, yielding 10 g/L of *A. oryzae* fungal biomass containing 47.6% protein [106].

#### 3.2.3. Food Industry Residues for *A. oryzae* Cultivation

Various industrial wastewater streams can serve as effective sources of organic compounds for supporting fungal biomass cultivation. Sar, Ferreira [95] reported that fish-processing wastewater, which is rich in minerals, fats, and proteins, can be used for the submerged cultivation of *A. oryzae.* The resulting biomass can be further used as an alternative protein source in fish feed. In addition, Sar, Ozturk [107] also reported that, using wastewater from an olive oil mill, protein-rich *A. oryzae* biomass can be cultivated (35 °C and pH 5.2). They reported that supplementing the wastewater with NaNO_3_ as the nitrogen source reduced the cultivation time from 90 h to 48 h [107]. Furthermore, Duru and Uma [89] used wastewater from cocoyam processing to cultivate protein-rich *A. oryzae* fungal biomass, finding that the addition of urea as the nitrogen source significantly enhanced the growth rate of the fungi. Considering wastewater as substrate, Jin, Yu [98] cultivated *A. oryzae* biomass, rich in 47% protein, using starch-processing wastewater for potential animal feed application. Wineries also generate a range of organic waste streams that can be managed by a reducing the organic load and producing value-added products. In a biorefinery approach, Zhang, Jin [93] used winery wastewater for producing *A. oryzae* fungal biomass with 36% protein. In addition, Jin, Zepf [99] grew *A. oryzae* fungal biomass on grape marc, achieving over 50% dry matter (DM) digestibility to be used for animal feeding.

Food-processing wastes are also a rich source of organics for the cultivation of edible fungal biomass. Hence, Yousufi [103] cultivated *A. oryzae* fungal biomass on different mixtures of fruit wastes and found that the maximum protein-rich biomass containing approximately 57% protein can be obtained by using a combination of pomegranate rind and guava peel, while a mixture of watermelon skin and apple wastes yielded the least (24% protein). According to Mahboubi, Ferreira [105], *A. oryzae* can be cultivated using dairy waste (expired milk, yoghurt, cream, etc.). Amylase secretion gives *A. oryzae* the ability to grow solely on oat flour to produce about 37% protein content that is of food and feed grade [94]. This growth capability has also been reported on substrates such as deoiled rice bran, with a final *A. oryzae* protein content of up to 43% [100]. VFAs are generated as intermediate metabolites of anaerobic digestion of organic waste. Effluents containing VFAs derived from mixed food wastes can also be used as the substrate for *A. oryzae.* This approach has resulted in the production of fungal biomass with 41% protein at a pH of 6 and temperature of 35 °C [83].

### 3.3. Nutritional Profile of Filamentous Fungi

From a nutritional perspective, fungal biomass contains fibrous polysaccharides, vitamins, lipids, minerals, proteins, and amino acids of particular interest required in ruminants’ diets [21].

Proteins are made up of amino acids and are an inseparable part of animal feed, responsible for structural and mechanical functions related to animal health, growth, reproduction, and/or lactation [112]. Nutritionally, the protein quality is evaluated based on the composition and bioavailability of amino acids, nitrogen content, level of harmlessness, and digestibility [113]. Amino acids are usually classified according to their role in the animal’s body and requirements in the diet. Amino acids supplied through the diet but not synthesized by the animal are considered essential or indispensable. All nine essential amino acids, including lysine, histidine, leucine, isoleucine, valine, methionine, threonine, tryptophan, and phenylalanine, are necessary to ruminants. Ruminants are provided with more amino acids through the digestion of microbial protein of ruminal microorganisms flowing into the abomasum and then the intestine. Amino acids that the animal can synthesize are often called non-essential or dispensable amino acids: glutamate, glutamine, glycine, serine, alanine, aspartate, and asparagine [112,114]. Amino acids in ruminants are used for microbial synthesis in the rumen, tissue maintenance and repair, and milk protein provision [112].

Filamentous fungi and yeast can have crude protein (CP) content up to 60% of their weight on a dry basis, mainly composed of arginine, lysine, and threonine. However, as is the case for soybean meal, fungal protein is poor in sulfur-containing amino acids such as methionine and cysteine, often limiting factors in ruminant diet inclusion [115]. Nevertheless, the composition of fungal biomass is dependent on many different factors as mentioned in previous sections. The proximate protein and amino acid composition of fungal strains *Neurospora intermedia*, *Aspergillus oryzae*, and *Rhizopus oryzae* compared to soybean meal, rapeseed meal, and fishmeal is presented in Table 2. According to a study by Karimi, Mahboobi Soofiani [84], fungal biomass contains a considerable amount of protein and amino acids comparable to soybean, rapeseed, and fish meals. Filamentous fungi were characterized by a CP content of 40–62%, with 30–36% (*w*/*w*) of this being essential amino acids. Considering their nutritional quality, all these species showed very high arginine, isoleucine, leucine, lysine, phenylalanine, threonine, and valine content. As can be seen in Table 2, CP and amino acid content varies between fungal species, which is also highly influenced by the growth media. For instance, the CP content of *A. oryzae* grown on baker’s yeast wastewater was between 43.8 and 55.5%, with 31.5% of CP being essential amino acids [102]. In turn, *A. oryzae* and *R. oryzae* utilizing waste streams from the pulping process contain 44–46% and 44% CP, respectively. The essential amino acids of both fungal biomasses were notably high, 53–55% CP, but were characterized by similar methionine and low lysine content compared to fish meal and soybean meal [86]. Jin, Yan [87] used starch-processing wastewater for fungal biomass production. The produced *A. oryzae* biomass contained more than 45% CP content with a noticeable quantity of essential amino acids up to 57%, with methionine at 2.45% and lysine at 15.54% as the most limiting amino acids in soybean, rapeseed, and fish meals [116].

Naturally, a fraction of proteins and amino acids supplied in the diet is immediately subjected to microbial degradation in the rumen. Microbial protein synthesis in the rumen supplies most of the amino acids available in the intestine [117,118]. This process makes it challenging to precisely predict how much protein and amino acids a ruminant absorbs. In this regard, meeting the amino acid requirements in ruminants depends on the availability of rumen-undegradable protein in the diet and the amount of amino acids from the microbial synthesis in the rumen [112]. However, the quality of protein in feedstuff is determined by the level of rumen-undegradable and rumen-degradable protein content.

From an animal feed perspective, filamentous fungal biomass contains lipids, fatty acids, and minerals. Fungal biomass contains more fat than the conventionally used protein source, soybean meal (Table 2). Although fungal biomass is not typically considered a lipid-rich ingredient for ruminant diets, it contains significant amounts of linoleic (C18:2), oleic (C18:1), and palmitic (C16:0) acids. Generally, the fatty acid provision in a ruminant’s diet improves the energy density of the ration and supports lactation and reproduction. Notably, polyunsaturated fatty acids such as linoleic acid (n-6) and linolenic acid (n-3) are known to improve reproduction in cows [112,119]. During fermentation, filamentous fungi can produce enzymes and other metabolites such as oligosaccharides, organic acids, and peptides which may have prebiotic effects on the animal [120]. These compounds can selectively stimulate the growth and activity of the beneficial gut bacteria in animals and exhibit antimicrobial effects [121]

The macromineral content in fungal biomass is directly influenced by the substrates used (Table 2). Vinasse [85] and baker’s yeast wastewater [102] contribute to the accumulation of calcium, potassium, phosphorus, magnesium, and sodium in *A. oryzae* and *R. oryzae* biomass. Additionally, synthetic glucose medium used by Karimi, Mahboobi Soofiani [84] and spent liquor used by Asadollahzadeh, Ghasemian [86] induced potassium and phosphorus accumulation in *A. oryzae* biomass, respectively. In general, fungal biomass can contain higher levels of minerals, mainly calcium, potassium, and phosphorus, than required in ruminants’ diets. Supplementing macrominerals in ruminants has been shown to improve average growth and reproduction. Macronutrients are structural components of bone and other tissues and are needed to maintain acid–base balance, osmotic pressure, electric membrane potential, and nerve transmission [112].

**Table 2:** Amino acid, fatty acid, and macromineral profile of *A. oryzae*, *N. intermedia*, and *R. oryzae* compared to soybean, fish, and rapeseed meal.

	Glucose			Vinasse			BYW	SSL60	SNL50	SSL60	POPE	SPW		SBM	RSM	FM
	AO	NI	RO	AO	NI	RO	AO	AO	AO	RO	AA	AO	RO			
Crude protein	45.7	62.2	50.6	44.7	57.6	50.9	43.8	44.4	46.3	44.9	39.6	45.7	49.7	43.3	33.7	31.8
Essential amino acids % CP																
Arginine	5.5	5.4	3.6	4.5	3.6	3.7	4.7	5.2	5.2	4.3	10.4			7.4	6.0	6.1
Histidine	1.9	2.12	1.8	1.7	1.5	1.5	1.5	7.5	6.3	8.0	3.6			2.7	2.6	2.5
Isoleucine	3.2	3.46	3.2	3.1	2.8	3.1	2.6	5.7	5.6	4.3	6.3	5.4	6.4	4.6	4.0	3.9
Leucine	5.3	5.95	5.0	5.5	4.7	4.8	5.7	8.7	8.3	8.1	8	8.8	7.8	7.4	6.7	7.1
Lysine	5.9	6.5	5.0	4.8	4.2	4.5	5.2	2.8	2.6	2.9	7.2	15.5	17.8	6.1	5.3	7.4
Methionine	1.2	1.5	1.3	1.3	1.1	1.2	1.0	1.5	1.9	2.9	1.5	2.4	3.1	1.4	2.0	2.6
Phenylalanine	3.2	3.3	3.1	3.2	2.7	2.9	3.6	3.8	3.5	3.7	5.2	11.1	9.4	5.0	3.9	3.9
Threonine	3.7	3.9	3.2	3.8	3.1	3.2	2.9	12.	12.4	11.2	4.8	4.6	5.7	4.1	4.3	4.1
Valine	4.1	4.4	3.6	3.8	3.5	3.6	4.0	7.9	7.8	7.7	7.1	4.6	6.2	4.8	5.0	4.8
Sum of AAs	34.2	36.5	29.9	31.6	27.3	28.4	31.5	55.2	53.6	53.2	54.1	52.4	56.5	43.5	40.0	42.6
Fatty acid content g/Kg DM																
Lipid %	6.9	4.0	17.2	7.0	5.5	3.5		11.4	7.4	5.7		1.9	1.1			9.8
Myristic acid C14:0	0.3	0.2	1.1	0.3	0.3	0.5		0.2	0.2	0.6	1.2			0.0	0.0	0.0
Palmitic acid C16:0	13.8	5.0	34.9	13.9	6.1	12.5		18.1	14.8	12.5	54.4			1.3	0.8	1.4
Palmitoleic acid C16:1	1.1	1.1	4.8	2.2	1.4	3.5		1.0	0.4	0.4	3.3			0.0	0.1	0.0
Stearic acid C18:0	4.1	1.2	8.8	5.4	1.8	3.4		6.0	3.7	4.6	3.7			0.5	0.3	0.8
Oleic acid C18:1	23.7	20.9	43.7	23.7	14.1	15.3		24.1	16.5	22.3	30.0			2.8	10.7	4.2
Linoleic acid C18:2	24.8	10.3	65.2	22.6	10.4	18.8		50.6	31.3	3.8	6.7			6.8	3.8	3.3
Linolenic acid C18:3	0.2	0.8	0.8	0.2	0.3	0.3		0.4	0.3	7.2				0.9	1.8	12.3
Macronutrient content g/kg DM																
Calcium	1.0	1.7	3.0	23.8	58.5	26.4	56.3	2.9	3.5	1.8		0.2	0.2	3.4	8.3	4.5
Potassium	11.3	9.3	1.3	12.0	15.8	8.9	9.3	11.2	12.8	7.4		1.4	0.5	21.2	12.3	10.3
Phosphorus	12.4	17.5	21.2	9.1	8.9	16.4	27.5	17.2	16.7	20.5		1.6	2.0	6.2	11.4	8.0
Magnesium	0.6	1.1	0.4	0.9	0.8	1.2	3.2	3.6	3.9	2.6				2.9	4.9	4.3
Sodium	0.3	0.3	0.3	1.1	1.3	1.6	3.4	4.5	4.8	3.2		0.4	0.2	0.0	0.4	1.0
Sulfur	4.6	4.7	3.1	4.7	4.8	3.9	4.6								5.9	3.6

AA: Amino acid, AO: *Aspergillus oryzae*, NI: *Neurospora intermedia*, RO: *Rhizopus oryzae*, DM: dry weight, CP: crude protein. AA was calculated and adapted based on the data of the following references: SSL: spent sulfite liquor and SNL: spent NSSC liquor [86], vinasse [85], BYW: baker’s spent liquor [102], POPE: palm-oil-processing effluent [122], SPW: starch-processing wastewater [87], SBM: soybean meal, RSM: rapeseed meal, and FM: fish meal [123].

## 4. Protein Digestion in Ruminants

Ruminants are herbivores categorized as forestomach fermenters. Their digestive stomach comprises four compartments: the rumen, reticulum, omasum, and abomasum, each of which playing a specific role in breaking down the ingested feed materials [124]. The rumen is the bulkiest and most complex compartment where fermentation occurs through the symbiotic activity of bacteria, protozoa, archaea, and fungi [125]. The rumen is connected to the reticulum by a large orifice and the digesta (ingested feedstuff going through digestion) move freely between them. The rumen and reticulum account for more than 50% of the total digestive capacity. During ruminal fermentation, fibrous feed is principally metabolized into volatile fatty acids, methane, carbon dioxide, as well as energy for microbial growth. VFAs contribute to more than 70% of ruminant metabolizable energy, with approximately 50–70% of the digestible energy being converted to VFAs, while the remainder is used for microbial growth and lost as gasses and urine [118]. If the protein content of the feed is digestible in the rumen, it is converted to amino acids and ammonia. The digested materials, including the solubilized carbohydrates, move from the reticulo-rumen to the omasum, usually with a particle size of 2 mm in diameter. The omasum walls are covered with short papillae that contribute to the reabsorption of water from digesta and further reduce digesta particle size. In the next chamber, the abomasum, which resembles the human stomach, the digesta are subjected to enzymatic digestion by pepsin and HCl before passing through to the small intestine.

Proteins are one of the primary essential nutrients in ruminants’ feed. However, the complexity of their degradation and digestion in the ruminant digestive tract makes it challenging to define the quality of protein required in feed. In general, ruminants rely on both dietary and microbial protein as their primary sources of protein. The quality of dietary crude protein is valued based on rumen degradability [124], as discussed in Section 2.

The rate and extent of protein degradation in the rumen vary depending on the type of protein and accessibility of peptide bonds, predominant microbes’ proteolytic activity in the ruminal microflora, and composition of the diet, particularly the energy supply provided by VFAs. Bacteria, which account for 50% of the microbial biomass, play a significant role in protein degradation due to their proteolytic activity [117,118,125]. Protein degradation in the rumen starts with the hydrolysis of peptide bonds by the proteolytic enzymes, producing smaller peptides and amino acids that are then deaminated into ammonia, energy, and CO_2_ [117]. The released amino acids and ammonia are primarily used for microbial protein synthesis. Proteolytic bacteria that cannot utilize the amino acids use ammonia as nitrogen and peptides as carbon and energy sources [118]. In addition to ammonia, amino acids, and peptides derived from RDP, the non-protein nitrogen (NPN), including nitrogen from nucleic acids (DNA and RNA), ammonia, urea, and small peptides, is directly used for microbial protein synthesis [117]. However, feeds with insufficient RDP decrease ruminal ammonia, reduce dry matter intake, and lower microbial protein production. On the other hand, feeds with excessive RDP enhance the production of ammonia nitrogen in excess, which is absorbed into the bloodstream, converted to urea in the liver, and excreted in urine [126]. Microbial protein synthesis is mainly influenced by the availability of nitrogen along with energy from fermentation carbohydrates, and branched-chain acids, supported by minerals [117,118].

The protein that escapes the ruminal degradation is either rumen-undegradable protein or bypass protein which does not degrade in the rumen, or indigestible protein which does not degrade in the ruminant body. RUP and microbial protein are enzymatically digested in the abomasum and absorbed in the small intestine for tissue growth, fetus protein, and milk protein [117,118]. Microbial protein accounts for 50–80% of absorbable amino acids, of which nearly 80% of RUP is absorbed. However, the amount of protein escaping microbial degradation in the rumen depends on the RUP source [117]. Ruminants generally use forage proteins poorly due to their high degradability in the rumen compared to processed grains and protein feed supplements [44]. RUP sources include silages (such as alfalfa silage and corn silage), plant sources (such as soybean meal, canola meal, and rapeseed), and animal sources (such as fishmeal) [44,127]. Numerous studies have evaluated the RUP value of feedstuffs, including grains and protein-rich dietary supplements [44,127,128,129,130,131]. However, due to the low RUP content, most protein supplements undergo heat treatment or chemical treatment to protect the protein from ruminal degradation and enhance the availability of absorbable amino acids in the intestine [132]. Examples of the treatment of feed protein are presented in Table 3.

Given the restrictions on using animal origin protein sources for ruminant feeding in the EU and the controversial nature of plant-based proteins, SCPs, including filamentous fungi, have been extensively investigated as potential alternative feed protein sources. Although the proximate analysis of most fungal biomass produced from a wide range of substrates has proven that it is comparable to soybean meal and fish meal, the ruminal degradation of these sources is not well documented in the literature and requires future research.

## 5. Single-Cell Protein in Ruminant Feed

Single-cell proteins are promising alternative nutrient sources that have the potential to play a role in conventional feedstuff replacement. Utilizing SCP as a protein source in animal feed is not a recent area of scientific exploration. Conventional fermented feed ingredients such as silage have high-value nutrition because of the rich content of microorganisms that result in good availability of essential amino acids and a decent protein quality [140]. The growth of microorganisms synthesizes proteins with supreme quality that can compete favorably with other conventional protein sources [141].

For feed inclusion purposes, the first thing is to guarantee the safety of SCP that is to be used in animal feeding. In this regard, toxicity, mutagenicity, carcinogenicity, pathogenicity, and organoleptic appropriateness are the factors to be considered. Although SCPs could be used as animal feed without additional processing, they should always be mixed with energy-rich feed ingredients such as corn, wheat, barley, and other grains, and this helps to have a balanced energy-to-protein ratio [142]. Nutritional and physiological values of microbial biomass, considering the actual concentration, digestibility, protein efficiency ratio (PER), net protein utilization (NPU), and biological value (BV), ensure an excellent balance in a ration comprising 5–20% single-cell protein [142,143].

Based on the composition of amino acids, vitamins, and nucleic acids, the nutritional quality of SCPs and whether their application can be beneficial or detrimental to animal well-being are defined. In this regard, although the rigid cell wall and high content of nucleic acids may be problematic, high-quality protein supplied with minerals, enzymes, and vitamins can elevate the feed quality [144].

Two of the most important microorganisms for producing SCP for animal feed are yeast and filamentous fungi [145]. Many yeast genera such as *Candida*, *Pichia*, and *Saccharomyces*, as well as filamentous fungi like *Rhizopus arrhizus* and *A. oryzae*, are commonly chosen for feed supplementation because they are considered non-toxic and non-pathogenic to animals. As pointed out, fungi can grow on a wide range of inexpensive carbon and nitrogen sources [146]. For example, *A. oryzae* can grow on cellulose and produce fungal biomass via fermentation. This biomass can further be used as a feed additive that assists and eases ruminal degradation and fermentation of cellulosic fibers. It has been proven that rations supplemented with *A. oryzae* improve beef calves’ performance by increasing dry matter intake and body weight gain [142], as well as increase milk production and reduce heat stress for dairy cows [142,143]. Additionally, *A. oryzae* fermentation extract has been reported to ameliorate the ruminal digestibility of forages with a high content of neutral detergent fiber [115]. Nucleic acids can constitute up to 10% of SCP weight. This nucleic acid content increases the uric acid concentrations in serum and urine [147], limiting the daily intake of SCP for monogastric animals such as chickens and pigs. However, ruminants such as cattle, sheep, and goats can withstand high levels of uric acid, as they are able to break down and excrete it as ammonia. Hence, the nucleic acid content of SCP would not be a limiting factor for dry matter intake in ruminants [148].

In recent decades, significant research has focused on the effect of SCPs on various aspects of ruminant health and performance, including dry matter intake, weight gain, temperature control, weaning age, milk production, dry matter digestibility, and the type and population of rumen microorganisms. To better understand the importance of SCPs in ruminant diets, Section 5.1 and Section 5.2 present an overview of the diverse effects of dietary SCP supplementation on ruminants.

### 5.1. The Effect of Aspergillus Oryzae Supplementation on Ruminal Digestion

Among feed additives, DFMs offer significant potential for modifying rumen fermentation by introducing viable naturally occurring microorganisms. While fibrous roughages, which make up 40 to 100% of ruminants’ basic rations, have low energy and nutrient density, DFMs can enhance their nutritional value. Physiological and economic reasons have led to an increase in the percentage of forage in the lactating cow’s diet. However, the low digestibility of plant cell walls in roughages and the low microbial yield in the rumen limit the conversion rate and efficiency of roughages into milk and meat [149]. Therefore, to enhance microbial protein synthesis and fiber digestibility, recent studies have extensively explored the manipulation of the rumen microbial community [64]. On the other hand, public concern over the use of antibiotics and growth stimulants in the animal feed industry has given rise to interest in the application of DFMs to replace antibiotics [150]. With DFMs, refinements in productivity could be attributed to changes in the rumen fermentation, distinct enhancement in forage degradability in the rumen, the flow of microbial protein from the rumen, and the numbers of viable bacteria that can be retrieved from the rumen fluid [151]. The possible effects of *A. oryzae*, *S. cerevisiae,* and other filamentous fungi on rumen fermentation are presented in Table 4. Inactivated microorganisms, along with their products and the cultivation medium, are categorized as fungal products (prebiotics). The enzymes in the fungal product convert macromolecular nutrients to available nutrients, stimulating the growth of rumen microorganisms [25,152].

#### 5.1.1. Effect on Dry Matter Digestibility

Yeast *S. cerevisiae* and filamentous fungi *A. oryzae* are the two most prominent DFMs in ruminant nutrition, often referred to as fungal feed additives or fungal prebiotics. *A. oryzae* culture (AOC) primarily enhances rumen fiber degradation, resulting in improved feed digestion [25]. AOC has been shown to positively impact various rumen microorganisms such as proteolytic, cellulolytic [169], and lactic-acid-fermenting bacteria [24], thereby increasing the digestibility of DM. Contemporary investigations have remarked that the disappearance rate of dry matter from Dacon bags [151] and in vitro fiber digestion [170] is accelerated by the administration of *A. oryzae* as a fungal feed additive. This enhanced digestibility, in turn, improves ruminant performance, such as higher milk production [171,172] and feed intake in cattle [173,174]. The digestibility rate, not the extent of digestion, of fiber fractions of bromegrass and switchgrass was also enhanced by the supplementation of *A. oryzae* [152].

Production responses to adding fungal supplements in feed have not always been consistent. Although Van Horn, Harris Jr [174] and Wiedmeier, Arambel [158] observed improved DM digestibility with the application of *A. oryzae*, several studies did not record any improvement in DM, organic matter (OM), cell wall components, and CP digestion with the addition of *A. oryzae* [175]. The response to the added fungal supplements appears to be diet-dependent and the discrepancies in the results could partly be explained by variations in feed composition [166]. The mentioned studies differed in the assortment of rations fed, varying from barley straw to fifty percent hay, with either corn or barley as a concentrate. Hovell, Nǵambi [176] described an interlocked association between the physicochemical structure of the forage, ruminal retention time, feed digestibility, and fungal supplement. There is little evidence that *A. oryzae* affects the cellular structure of plant tissue; this would explain why several studies have found that fungal additives do not affect digestibility. In ruminant feed supplementation, both changes in the ruminal fermentation and response to the additive inclusion are affected by the ration. Huber, Higginbotham [177] uncovered that the reaction to *A. oryzae* in lactating cows was more substantial on a low-forage diet than on a high-forage diet. Multiple studies have demonstrated that *A. oryzae* improved NDF digestibility of switchgrass and bromegrass at 12 h [152] and alfalfa hay at 48 h [178] and DM digestibility of a total mix ration (TMR) at 24 h in vitro [170]. Positive outcomes from the animal experiment also revealed that DM and CP digestibility were enhanced by this fungal supplementation [158], enriching the digestibility in the total tract. However, the microbial protein flow from the rumen was incited by both low- and high-forage diets [77]. It is conceivable that only the cows on the low-forage ration had adequate energy available to benefit from the supplemented protein flow, albeit the same increase would ensue both rations.

Raper and Fennell [179] authenticated the cellulolytic, amylolytic, proteolytic, and antibiotic activity of *A. oryzae* fermentation extract (AFE), which would ameliorate fiber digestibility in lambs receiving a ration containing mixed vetch and oat hay, maize silage, and barley meal [163]. However, the inconsistent results from Firkins, Weiss [180] indicated that *A. oryzae* had little influence on the digestion of orchardgrass hay by Holstein heifers. In a recent study by Uwineza, Bouzarjomehr [181] on *A. oryzae* fungal biomass produced from volatile fatty acids derived from agro-industrial residuals, they suggested that the higher ruminal digestibility of fungal biomass would contribute to the rumen digestion.

#### 5.1.2. Effect on Rumen Microbiota

One of the various microbial feed supplements commercially produced is Amaferm^®^ [182], a fermentation extract of *A. oryzae* that acts as a prebiotic, enhancing the population of ruminal fungi. Fungal mycelium contains bioactive compounds, and cell wall compounds facilitate the release of bacterial enzymes, increasing nutrient digestion in the rumen. Furthermore, the mycelium and cell wall compounds increase microbial protein volatile fatty acid production and stabilize the pH of the rumen. As per studies, the ruminal microbial activity in a rumen simulator technique, Rusitec [76], and cows’ rumen [158] was increased by adding *A. oryzae* to feed due to the enhancement of VFA concentrations and augmented bacterial counts. Notably, fiber-digesting bacteria and lactate-fermenting bacteria were the two bacteria reacting positively to *A. oryzae* supplementation.

*Fibrobacter*, *Succinogenes*, and *Ruminococcus albus* are among the prevailing fibrolytic bacteria in the rumen of cattle and sheep [24]. The results of in vitro and in vivo experiments have shown that *A. oryzae* supplementation in feed enhances the growth rate of these bacterial species [76,183]. In addition, *A. oryzae* may retain some endogenous proteolytic activity [184] that could enrich the concentration of branched-chain VFAs [76]. The growth rate of many ruminal fibrolytic bacteria is affected by branched-chain VFAs and bioactive compounds [185,186]. Wiedmeier, Arambel [158] reported that adding 2.3 g/day of *A. oryzae* to the diet improved the total bacterial count, especially cellulolytic bacteria, by 27%. Frumholtz, Newbold [76] discovered an 80% boost in the total and 188% growth in the cellulolytic bacterial population in vitro augmented with 250 mg/day *A. oryzae* supplementation. In compliance, the numbers of cellulolytic, hemicellulolytic, and pectinolytic bacteria grew in calves fed by *A. oryzae* [183]. In contrast, Fondevila, Newbold [4] recorded no upsurge in the cellulolytic bacterial population, while a 126% increase in the bacterial count of sheep supplemented with 2 g/day *A. oryzae* was observed. A more dynamic population of fibrolytic ruminal bacteria is expected to enhance fiber degradation; however, the results regarding digestibility along with *A. oryzae* supplementation have been rather controversial. Wiedmeier, Arambel [158] reported an improvement in the total tract digestibility of hemicellulose, but not acid detergent fiber (ADF), in response to the addition of *A. oryzae* to the diet. On the contrary, Frumholtz, Newbold [76] noted that *A. oryzae* had no impact on in vitro DM digestion, having little effect on cellulolytic bacteria counts.

Even though *A. oryzae* supplementation is said to increase the cellulolytic bacterial numbers [187,188], the rate and extent of fiber digestibility do not improve correspondingly. Evidence supporting this hypothesis came from *A. oryzae*’s extensive enzymatic activities, including carboxymethylcellulase activity (CMCase) [157], which can potentially facilitate fiber digestion. Although *A. oryzae* does not depolymerize structural carbohydrates thoroughly to simple sugars by its enzymatic machinery, it does generate enzymes that induce unfinished depolymerization [189] and assist rumen cellulolytic bacteria in depolymerization of cellulosic material [190].

*Selenomonas ruminantium* and *Megasphaera elsdenii* are common Gram-negative ruminal lactate-utilizing bacteria. They can constitute up to 51% of the total viable ruminal bacterial count [191], especially in cattle that are fed a high-concentrate diet [192]. *Megasphaera elsdenii* generates the branched-chain VFAs and ammonia that are essential for the growth and activity of cellulolytic bacteria. The cellulolytic bacteria, in turn, supply the required soluble carbohydrates for lactate-fermenting bacteria [185]. Previous studies revealed that aspartate, carbon dioxide, p aminobenzoic acid, and biotin are the prerequisites for their growth in a lactate-salts medium [193,194]. Additionally, L-malate and fumarate could surrogate aspartate in the medium. *A. oryzae* produces fumarate and malate as they existed in considerable quantities in all strains tested [195,196]. The involvement of malate as a stimulator could increase the lactate utilization by *S. ruminatium* and *M. elsdenii* treated with *A. oryzae*. In addition, *A. oryzae* supplies growth factors, such as amino acids and B vitamins, plus enhances lactate uptake by the lactate-utilizing bacteria. Nisbet and Martin [196] indicated a higher growth rate and shorter doubling time in medium containing *A. oryzae* for *S. ruminantium*. In addition, Waldrip and Martin [197] reported the stimulation effect of *A. oryzae* on the *M. elsdenii* growth rate.

VFAs are produced via microbial fermentation of carbohydrates in the rumen; therefore, increased ruminal VFA concentrations are frequently considered a consequence of enhanced fermentation and better digestibility. Different types of rations have different chemical compositions that alter the response of *A. oryzae* to the microbial population in the rumen. For instance, *A. oryzae* supplementation to a diet with a high level of soluble carbohydrate veers fermentation towards more propionate production [178]. Supplementation of *A. oryzae* results in a versatile response in the acetate to propionate ratio [158,184,198]. Beharka and Nagaraja [24] found that the acetate concentration in the medium from *R. albus* increased with the addition of 5% *A. oryzae* to the ration. In contrast, the same concentration of *A. oryzae* supplementation increased propionate produced by *S. ruminatium* in the medium without affecting acetate production, leading to a decreased acetate to propionate ratio. The production of propionate competes with methanogenesis for available hydrogen, resulting in lower methane production. Supplementing with *A. oryzae* stimulates propionate-producing bacteria such as *S. ruminatium*, *Ruminococcus albus*, and *Librobacter succinogenes*, creating a hydrogen sink that limits methanogens, thereby reducing methane formation. This shift in rumen microbial diversity redirects metabolic hydrogen from methane to propionate production, resulting in lower methane and overall gas output [199,200]. Counotte, Prins [201] reported butyrate production by lactate-utilizing bacterium *M. elsdenii* and lactate-utilizing bacteria to keep ruminal pH under control by constant removal of lactic acid [202]. It is noteworthy that butyrate has growth-promoting effects on rumen epithelial cells [203,204]. The addition of 2% *A. oryzae* to a culture containing *M. elsdenii* boosted the production of total VFAs and increased butyrate from 6.3 to 7.2 mM.

The addition of more grains and cereals to the ration and increasing the concentrate/forage ratio would provide more energy for ruminants but it has a negative effect on structural carbohydrate digestibility and eventually alters the cellulolytic bacteria habitat [205]. To maintain the digestibility equilibrium at an efficient level, two strategies should be employed. One is feeding buffers to keep an optimal ruminal pH of 6.7 to 7 [206] and the second is to increase structural carbohydrate digestion in high-grain diets. *A. oryzae* as a fungal additive in feed could facilitate both approaches. It could increase lactate uptake by the ruminal bacteria *S. ruminatium* [196] and *M. elsdenii* [197] to maintain rumen pH at the optimum level and, therefore, provide conditions for enhancing the activity of fibrolytic bacteria in high-cereal-grain diets [76,77].

Aside from the stimulatory effect of *A. oryzae* on fibrolytic bacteria and lactic-fermenting bacteria, supplementation of *A. oryzae* inhibits the ciliated protozoa population that prey on bacteria, which may partially account for the enhanced bacterial counts [157]. Frumholtz, Newbold [76] noted that the addition of AFE to the rumen in an in vitro test using Rusitec^®^ reduced the protozoal population by 45%. Therefore, defaunation would allow an increase in the bacterial community. In a study by Niver, Tucker [163], adding *A. oryzae* to wethers’ feed eliminated the protozoa from the rumen within two weeks after initiating the treatment. However, Oellermann, Arambel [207] reported minor changes in ruminal protozoa counts by *A. oryzae* supplementation.

The effects of *A. oryzae* may not be restricted to bacterial growth enhancement, as Gomez-Alarcon, Dudas [77] found that *A. oryzae* has proteolytic activity. Feeding *A. oryzae* increased the NH_3_-N concentration in the rumen, an indication of higher protease activity. Deamination of amino acids has also been reported to increase by *A. oryzae* supplementation [182]. Wiedmeier, Arambel [158] found that *A. oryzae* promoted ruminal crude protein digestion in situ, with branched-chain VFAs being the products of this protein degradation.

Therefore, fungal additives in TMR would increase the bacterial diversity that may grow and consume NH_3_-N to produce more microprotein. This hypothesis may explain diminished NH_3_-N concentration in TMR [178,182]

### 5.2. Effect of Aspergillus Oryzae Supplementation on Ruminant Products

During recent decades, fluctuations in feed prices have caused adversities for dairy cow producers, resulting in a reduction in profit margins. Factors such as shortages of resources and environmental challenges such as drought or storms may affect feed prices and dairy producers’ profitability [65,208].

Therefore, nutritionists have been looking for new technologies and feed additives to enhance the quality and efficiency of feed while minimizing residual effects. These advancements aim to enhance ruminant product yield from feed, thereby increasing value creation from less conventional feedstock. The application of feed supplements and additives such as herbs [209,210], phytogenic extracts [211,212], exogenous enzymes [213], and DFMs [63,214] has made progress in in vitro and in vivo experiments. Recently, filamentous fungi, yeast live cells, and cultures have been marketed as non-bacterial DFMs for lactating cows’ feed supplements [215]. The dominant species from which these cells and cultures originated are distinct strains of *A. oryzae* and *S. cerevisiae* [216]. The mode of action of *A. oryzae* and *S. cerevisiae* as feed additives is not thoroughly understood, as they are not metabolic products with quantifiable molecular structures like antibiotics. Nevertheless, this has led researchers to speculate on the effectiveness of *A. oryzae* and *S. cerevisiae* as feed additives for ruminants [217]. The potential of *A. oryzae* as a feed additive in ruminant performance is presented in Table 5.

The effect of *A. oryzae* fermentation product supplementation on milk yield and composition in ruminants is rather diverse. Yu, Huber [218] reported a tendency to enhance milk protein and solids-not-fat (SNF) content by the inclusion of AFE in the diet of lactating cows. On the other hand, Bertrand and Grimes [153] recorded no positive response in milk production with AFE inclusion. However, the effect of AFE on milk yield and milk constituents could be influenced by various factors including the quality of AFE, feed composition, and environmental conditions [219].

Waldo and Jorgensen [220] reported that 50 to 75% of the variability in lactating cows’ productivity could be attributed solely to differences in feed intake. Supplementation with AFE or *S. cerevisiae* can increase feed intake, enhance nutrient digestibility, and boost ruminal fermentation activity, leading to improved milk production [26]. Interaction between the addition of *A. oryzae* and animal maintenance factors such as feed composition, environmental conditions, and animal health would change the animal’s response to fungal supplementation in their diet. This interaction would result in variations in milk production and differences in milk composition. As Sucu, Moore [221] added 15 g AFE to the daily ration of lactating dairy cows they observed an increment in milk production.

Both feed components and environmental factors can significantly affect an animal’s responses to the supplementation of fungal additives. Rations including more non-fibrous carbohydrates boost AFE effectivity on dairy cows more than rations high in fiber [222]. Some studies imply that AFE as a supplement for enhancing milk yield and constituents was more restrained in high-energy or low-fiber diets. Wallentine, Johnston [172] and Gomez-Alarcon, Wiersma [223] proposed that the effectiveness of AFE on milk yield and constituents could be influenced by the diet composition and ratio of concentrate to roughage. However, when Yu, Huber [218] investigated the effect of AFE addition with steam-flaked or steam-rolled corn in the diet, they observed that despite an increase in the concentrate ratio in the diet, there was no positive effect on milk yield and components.

Chiou, Chen [217] investigated the correlation between feed composition and season in an experiment with lactating dairy cows. They found that feed with high energy content, such as corn silage supplemented with AFE, improved dry matter intake and milk production in the hot season. However, the alleviation in milk production was more meaningful in the cold season, although not followed by an increase in dry matter consumption. Furthermore, a combination of AFE with silage could increase soluble protein and NPN in silage. These high soluble protein amounts may provide a surplus fraction of ruminal ammonia that transcends the capacity for bacterial ammonia removal sufficiency, resulting to increased blood and milk urea concentrations [224]. This surplus is partly due to the increment in deamination and protein digestibility in the rumen [158,225]. Moreover, since the production of ruminal acetate, which is a precursor for fat biosynthesis, could be enhanced by improved fiber digestibility, it is assumed that AFE addition, which benefits fiber digestibility, should increase milk fat content [166,226]. However, although the underlying reasons were not fully clarified, Sallam, Abdelmalek [26] reported a decrease in milk fat concentration with AFE addition.

The animal lactation period could alter with the interaction between ration composition and fungi supplement inclusion. Adding AFE to rations in different lactation periods might produce different milk yield, fat, true protein, and SNF responses. A study comparing the effect of AFE inclusion between the early and late stages of the lactation period observed that AFE promoted milk production and raised the mean fat-corrected milk (FCM) to about 3.5% through the early stages of the lactation cycle of dairy cows [171]. High-concentrate rations such as corn silage as basal diet supplemented with AFE at 56 g/d resulted in increased milk production and milk fat content in the early and middle stages of the lactation cycle of dairy cows [173]. Additionally, Huber, Higginbotham [177] showed that adding 90 g/d AFE in the early lactation period from 1 to 90 days would enhance milk production from 18.5 kg·d^−1^ to 20.2 kg·d^−1^. According to Gomez-Alarcon, Huber [166], AFE inclusion would elevate dry matter intake, fiber, and crude protein digestion, leading to higher milk production in early lactation. In confirmation of this study, Kellems, Lagerstedt [171] also reported the highest milk production during the early lactation period while AFE was supplemented. In contrast to these studies, Higginbotham, Santos [227] observed only an improvement in milk protein content and no other modifications in lactation efficiency of early-lactation cows when AFE was added to feed. Results from this study supported an earlier investigation by Denigan, Huber [162] that feeding AFE at more than 3 g/d had no specific effect on milk yield.

Published data representing the effects of *S. cerevisiae* on milk production are minimal [228,229]. However, it has been reported that the addition of *S. cerevisiae* with AFE to a high-concentrate ration improves milk production associated with feed intake increment [228,229]. Several mechanisms could explain the body’s responses to the supplemented ration with fungal additives in terms of milk yield and composition. The energy required for maintenance and production in the body may come from the mobilization of body reserves or be provided through increased amounts of energy and/or protein in the diet [228].

Besides improving milk yield and composition of dairy cows, *A. oryzae* supplements positively impact dry matter intake in dairy and beef cattle. As mentioned earlier, AFE enhances rumen microbial activity, improves forage digestibility, and increases the flow rate, all of which contribute to increased feed intake [230]. Beef cattle gain weight more quickly when their feed intake is increased, and feed conversion ratios improve. Tricarico, Abney [231] added *A. oryzae* extract containing α-amylase to two types of processed corn (dry cracked and high moisture). By both processing methods, supplementation improved average daily weight gain during the initial 28 days in 120 crossbreed steers. Contrary to this, Zerby, Bard [232] observed no difference in carcass characteristics between high-moisture corn and dry whole-shelled corn supplemented with AFE. Dry whole-shelled corn supplemented with AFE led to an improved feed conversion ratio, attributed to a 7.9% reduction in dry matter intake. Evidence of direct physiological effects from fungal additives on increasing the mobilization of body reserves is scant.

**Table 5:** The effect of added *A. oryzae* as feed additive on the performance of ruminants.

Figure	Concentration	Form of Addition	Subject Animals	Variables	Findings	Ref.
AO	3 g/d	Added with 5.6% tallow	28 Holstein cows	Milk production, dry matter intake	Did not stimulate any variables.	[153]
AO extract, Amaferm^®^	1 g/d	Supplemented into 85% concentrate diet	48 lambs	Growth and carcass characteristics	ADG increased.	[232]
SC with AO	0.5 g/d SC plus 3 g/d AO	Whole-shelled corn or high-moisture corn	48 lambs	Growth and carcass characteristics	BW increased.	[232]
SC with AO	10 g/d SC plus 3 g/d AO	Supplemented into diet (60% rolled barley and 40% timothy hay)	40 dairy cows	DMI and milk yield and composition	Higher daily milk production, better weight gain.	[25]
AO	3 g/d	Inclusion in silage and TMR	7 dairy cows	Milk yield and composition	Increased yield, 4% FCM.	[217]
AO and/or SC	3.5 g/d	Supplemented into the basal diet	80 multiparous lactating cows	Lactation performance	Increased feed intake and daily milk production.	[26]
AO	113 g/day	Supplemented into the basal diet with 35% whole cottonseed	108 Holstein cows	Milk production	Decreased milk production and feed intake.	[174]
AO culture	3 g/d	Supplemented into steam-flaked and steam-rolled corn	32 multiparous Holstein cows	Milk yield and compositions	Increased protein and SNF percentage.	[218]
AO extract	3 g/d	Supplemented into the TMR plus 136 g of rice mill by-product	110 multiparous lactating cows	Rumen metabolites and milk production	Increased SNF percentage, lower blood urea N concentrations.	[216]
AO extract	5 g/day	Supplemented into the TMR	282 multiparous Holstein cows	Milk production and composition	Lesser concentration and yield of milk true protein compared to control group.	[227]
AO extract with yeast culture	56 g/d (yeast culture) plus 3 g/d (*A. oryzae*)	Supplemented into the TMR	521 Pluriparous Holstein cows	Milk yield and composition	Lower percentages of lactose and SNF.	[233]
AO extract containing α-amylase activity	12 g/d	Supplemented into the TMR	150 lactating cows from 45 commercial dairy herds	Milk production and composition	Slight milk yield increase, lower milk fat percentage.	[234]
AO fermentation extract	15 g/d	Supplemented into the TMR (alfalfa hay and steam-flaked corn)	33 Holstein cows (22 multiparous and 11 primiparous)	Productive variables in transition dairy cows	Increased milk production, decreased plasma non-esterified fatty acids.	[221]
AO fermentation extract	15 g/d	Supplemented into the TMR (corn silage and rolled corn)	2455 multiparous Holstein cows	Milk yield and compositions	Decreased milk yield, increased milk fat content.	[221]
AO fermentation extract	3 g/d	Supplemented into the TMR	210 early-lactation Holstein cows	Milk yield and compositions	Increased milk production, increased FCM 3.5%.	[171]
AO fermented culture	10% (*w*/*w*) levels	Added to a commercial concentrate (GT-03)	15 Garut sheep	Dry matter intake	Increased protein intake and organic matter.	[235]
AO fermentation extract	1.5 g/d	Supplemented into the TMR	64 lactating cows	Digestibility of CP, NDF, and DM	Increased DMI.	[162]
AO fermentation extract non-ionic surfactant	100 g/d (prepartum) 150 g/d (postpartum)	Supplemented into the TMR	40 Holstein dairy cows	Dry matter intake (DMI) and milk production	Greater DMI in the transitional period, increased milk fat content.	[164]
AO fermentation extract	2 g/d	Top-dressed on texturized starter ration	52 bull calves	Growth rate at weaning age and rumen development	Tended to increase ADG.	[165]
AO culture	3 g/d	Supplemented into the diet (70% concentrate and 30% forage)	2 dry and 2 lactating cows	Nutrient utilization	Increased rumen and total tract digestibility of fiber fractions.	[77]
AO fermentation extract	3 g/d	Top-dressing at the morning feeding plus 87 g of ground sorghum	46 lactating cows (20 primiparous and 26 multiparous)	Milk production and composition	Increased milk production.	[166]

Abbreviations: *Saccharomyces cerevisiae* (SC), *Aspergillus oryzae* (AO), total mixed ration (TMR), fat-corrected milk (FCM), solids not fat (SNF), dry matter intake (DMI), average daily gain (ADG), body weight (BW).

## 6. Perspectives for Circular Bioeconomy

Single-cell proteins are already considered a potential feed additive to supplement ruminant protein. Incorporating fermented fungal biomass, particularly *A. oryzae*, in ruminant diets offers a sustainable environmentally friendly approach to livestock farming. This practice holds significant promise for reducing greenhouse gas emissions, primarily methane, and enhancing feed efficiency, contributing to climate change mitigation and environmental sustainability. Supplementing ruminant diets with *A. oryzae* fungal biomass can enhance feed digestibility, leading to better nutrient absorption and a shift in bacterial populations that could inhibit methanogens, thus reducing methane emissions. In addition, valorizing nutrients from agriculture residues, by-products, and waste streams as substrates for fungal fermentation reduces the need for disposal and mitigates the environmental impact of waste residuals. This approach aligns with the circular bioeconomy framework, contributing to sustainable ruminant farming.

Although fermentation extracts of *A. oryzae* and other microorganisms like *S. cerevisiae* are already commercially available and well documented, educating farmers, veterinarians, and technicians about the benefits and legalities of innovative feeding practices such as the use of fermented fungal biomass from agriculture residues is crucial for advancing sustainable livestock farming. Effective teaching methods can ensure these practices are widely adopted and properly managed. Appropriate regulations can facilitate the adoption of sustainable farming. Since the selection of microorganism strains used in processed feed additives is already subject to strict regulation, appropriate regulations that promote the use of agricultural residues and support research into advanced fermentation technologies are essential.

Future research and development on SCP for ruminant feed focuses on identifying versatile microorganism strains that efficiently convert agricultural residues and wasted food into high-protein biomass, optimizing fermentation processes and growth conditions through advanced techniques to boost protein content and nutritional value, and integrating artificial intelligence (AI) and precision fermentation to improve the productivity. Additionally, future research needs to assess environmental and economic sustainability through life cycle assessments and cost-effective methods, comparing the sustainability of SCP to traditional protein sources and the use of locally available substrates to make SCP more accessible and affordable for farmers. Further research is needed on microbial protein biomass from residual materials to evaluate its impact on ruminant production and feed efficiency.

This review focused on *A. oryzae*, one of the alternative protein-rich animal feed supplements and the most studied and promising filamentous fungus for application on an industrial scale. For years, many scientific studies have focused on the application potentials of filamentous fungi and their extracts and metabolites in ruminant feed applications at a laboratory level and in in vivo and in vitro experiments. Although *A. oryzae* fermentation extract has received much attention, its direct individual effect as a feed additive or ingredient is controversial; however, it works efficiently in synergy with other supplements, such as yeast. Because most results are controversial, there remains a gap in the accumulated knowledge and farming processing for using it as a feed supplement or a partial replacement with conventional ruminant ingredients. Therefore, further research is needed on microbial protein biomass derived from residual materials to evaluate its impact on ruminant production and feed efficiency.

## 7. Conclusions

Using the filamentous fungus *A. oryzae* to produce value-added proteins and nutrients from its fermentation using organic residues is a promising, sustainable approach for feed ingredients. Studies on the use of SCP, particularly filamentous fungi, such as *A. oryzae*, as feed supplements for ruminants have been conducted for decades. These studies consistently report positive effects of *A. oryzae* on ruminal digestibility and animal performance, such as fiber digestibility, dry matter intake, milk production, and body weight gain. Collective findings from long-term research on the inclusion of mainly *A. oryzae* filamentous fungal biomass in ruminant diets demonstrate its potential to enhance sustainability in ruminant production, with significant ability to reduce methane emissions and improve production efficiency.

## Figures and Tables

**Table 1:** *Aspergillus oryzae* cultivation on a variety of substrates for production of fungal protein.

Substrate	Substrate Loading	pH	Temperature (°C)	CP%	Working Volume	Main Findings	Ref.
Synthetic medium VFAs	3–18 g/L of mixture of acetic propionic, butyric, and caproic acid	5–8	35	<41	250 mL	The fungal growth inhibition increased with increasing acid concentration.	[83]
Winery wastewater treatment	50 mL	4–6	30	35.3	250 mL	To obtain the protein-rich fungal biomass, the biorefinery can be used in winery waste streams.	[93]
Oat flour	20 g/L oat flour, 10 g/L sucrose, and 100 mL cooking oil	5	35	37	26 L	-	[94]
Fish processing wastewater	3 L salt brine	5.5	35	54	4.5 L	*A. oryzae* has the potential to be cultivated on different wastewater streams.	[95]
Brewer spent grain	14.6 g of wet BSG	5.5	35	30.52	250 mL	The hydrothermal pretreatment can increase both solubilization of the substrate and the protein content of the produced biomass.	[96]
Black liquor	-	5.5	35	47.6	4 L	Compared to other fungal strains, *A. oryzae* has the highest yield of production using black liquor.	[86]
Vinasse	21 g/L for 5% vinasse solution	5–6.5	35	44.7	250 mL	Vinasse which is diluted 95% (*v*/*v*) supports the highest growth rate.	[85]
Agro-industrial residuals	14 g/L agro-industrial by-products and 11.5 g/L whey powder	5.5	24	-	500 mL	The optimum volume of free air must be about five time higher than the volume of medium to achieve the highest rate of production.	[97]
Glucose	30 g/L glucose	5–6.5	35	45.7	250 mL	-	[84]
Starch-processing wastewater (SPW)	-	5.2	38	46.7	-	Semicontinuous process of fungal cultivation using SPW has a higher productivity.	[98]
Grape marc residue	15 g of dried grape marc residues	-	30	23	250 mL	The produced biomass can be a good feed supplement since it has more than 50% dry matter digestibility.	[99]
Wastewater of cocoyam	200 mL	4.5	35	-	500 mL	By the addition of urea, the protein level of the produced *A. oryzae* biomass will increase about two times.	[89]
VFAs from food waste and cow manure	50 mL	6	35	Up to 41	250 mL	Using VFAs produced from food wastes as the substrate, protein-rich biomass production can be achieved.	[83]
Thin stillage	50 mL of medium	5.5	30–45	48	250 mL	*A. oryzae* can be preferable for cultivation on thin stillage compared to *S. cerevisiae* since it can consume pentose sugars.	[90]
Deoiled rice bran	10 g	3–7	36–45	43	500 mL	-	[100]
Soybean husk and flour mill	1 g	6	30	10	Solid state fermentation	-	[101]
Baker yeast wastewater	50 mL	5.3	30	43.8	250 mL	Nitrogen supplementation increases the protein content by 12%.	[102]
Wheat kernel	-	-	30–35	-	SSF	For obtaining a higher biomass yield, pretreatment is essential.	[91]
Fruit wastes	-	-	28	57.3	-	*A. oryzae* fungal biomass grows optimally on pomegranate rind and pineapple skin.	[103]
Whole stillage	1 L	5	35	42.3 ± 1.7	2.5 L	-	[104]
Dairy wastes mixture	100 mL of medium	4–7	35	30–40	250 mL	High concentration of lactic acid does not have a negative impact on degradation of fat by *A. oryzae.*	[105]
Paper pulp	-	6–9	30	-	500 mL	-	[106]
Olive oil mill wastewater	50 mL of olive oil mill wastewater	5.2	35	32.3	250 mL	Addition of nitrogen source decreased cultivation time and increased biomass protein content.	[107]
Glucose	30 g/L glucose, 5 g/L yeast extract	5.5	35	47	250 mL	*A. oryzae* has the potential to produce fungal biomass with high L-carnitine content.	[108]
Pea-processing by-product (PpB)	3.5 L PpB dissolved in distilled water	6.1–6.5	35	43.1	4.5 L	PpB can be applied for the production of protein-rich biomass.	[109]
Leaf and stalk	-	5	30	-	150 mL	-	[110]
Food industry by-products	-	6.5	30	58.9	-	-	[111]

**Table 3:** Treatment methods applied for increasing the RUP fraction of different feed protein sources.

Treatment Methods	Protein Sources	Treatment Conditions	Digestibility Treatments	Effect	Ref.
Heat treatment	Rapeseed meal (RM)	Mixed with water and heated at 110 °C for 30 min	In situ and in vitro intestinal analysis	RUP increased from 262 g/kg to 431 g/kg.	[133]
Moist heat pressure	Canola meal	Heat at 127 °C with steam pressure of 117 kPa for 15, 30, 45, 60, and 90 min	Nylon bag, mobile nylon bag, in situ and in vitro techniques	Nitrogen disappearance declined in the rumen from 74.4% to 18.9% and increased in GI tract from 16.2 to 64.2% for control and CM heat treated for 45 min.	[134]
Heat treatment	Soybean meal and fish meal	Heat at 120 °C and 130 °C for 30 min	Rumen degradation and CP partial digestibility in the digestive tract	RUP increased in TSBM 120 and 130 from 36.6 to 67.8 and 71.7%, respectively.	[135]
Extrusion	Dehulled lupin (DL) and RM	Heat at 130 °C with 20% moisture for DL and 120 °C with 20% moisture		RUP increased in EDL by 8.9% and 37.35% in ERM.	[136]
Fat coating	Soybean meal	Coat with 10 and 25% long-chain fatty acids (palmitic acid (PA) and stearic acid (SA))	In situ rumen degradation using nylon bag	RUP increased 76.57% to 82.47% and 90.92% in 8 h rumen degradation for 10% and 25% fat-coated soybean meal, respectively.	[137]
Fat coating	Soybean meal	Coat with 400–500 g fat/kg at different ratios of PA and SA	In situ rumen incubation and in vitro intestinal protein digestibility	RUP increased from 262 g/kg to 308 and 364 g/kg for FL40 and FL50, respectively, at 100% PA coating.	[133]
Lignosulfonate	Canola meal and soybean meal	Added 7% LSO_3_ and heated at 95 °C for 1 h	In vitro and in situ digestibility	RUP increased from 41.9% to 65.3% and from 30.9% to 63.3% in treated soybean meal and canola meal, respectively.	[138]
Formaldehyde	Canola oil cake meal and sweet lupin seed	Added 40% (*w*/*v*) formaldehyde at concentration of 10 g/kg and 15 g/kg	In situ dry matter and crude protein digestibility	The CP effective degradation was decreased from 71.7% to 38.1% at formaldehyde concentration of 15 g/kg.	[139]

RUP: rumen-undegradable protein; EDL: extruded dehulled lupin; ERM: extruded rapeseed meal; PA: palmitic acid; CP: crude protein; SA: stearic acid; GI: gastrointestinal; TSBM: treated soybean meal; CM: canola meal.

**Table 4:** The effect of fungal ruminant feed additives on rumen performance and feed digestibility parameters.

Fungal Additive	Concentration	Form of Addition	Experimental Approach and Subject Animals	Variables	Effects	Ref.
AO	3 g/d	Added with 5.6% tallow	28 Holstein cows	Neutral detergent fiber	Did not stimulate any variables.	[153]
AO and AN	30 mg	Supplemented into 500 mg of TMR, corn silage, oat hay, and alfalfa hay	In vitro analysis	Gas production, DM, CP, ADF, NDF	Significantly increased in gas production, DM, CP, ADF, and NDF for all rations.	[22]
AO and AN	30 mg	Supplemented into oat hay and alfalfa hay	In vitro analysis	Molar proportion of acetate and acetate to propionate ratio	The proportion and ratio of acetate:propionate was increased.	[22]
AO and AN	30 mg	Supplemented into TMR, oat hay, and alfalfa hay	In vitro analysis	Bacterial community	Increased *Prevotella* and decreased *Ruminococcus* counts.	[22]
AO and SC	10 g/d SC plus 3 g/d AO	Supplemented into diet (60% rolled barley and 40% timothy hay)	8 steers	Ruminal fermentation and bacterial counts	Higher concentrations of acetate, propionate, and total VFAs tended to increase ruminal NH_3_-N concentration and decrease pH.	[25]
AO	2 g/d	Supplemented into diet (grass hay and barley)	4 sheep	Ruminal fermentation and bacterial counts	Reduction in lactate and propionate in rumen.	[154]
AO	2 g/d	Supplemented into chopped barley straw (plus urea and minerals)	8 sheep	Digestibility parameters and bacterial counts	Increased initial rate of feed degradation and total bacteria counts.	[151]
AO and/or monensin	500 mg/day	Supplemented into the diet (hay, barley, molasses, fish meal, and mineral/vitamin mix)	In vitro analysis (Rusitec)	Fermentation parameters, microbial community	Increased propionate and reduced butyrate, increased bacterial count, and non-significant reduction in protozoal numbers.	[155]
AO extract with alpha-amylase activity	450 FAU/kg DM	Supplemented into the basal diet	24 multiparous Holstein cows	Total tract digestion, ruminal fermentation, nitrogen utilization	Increased CP and DM digestibility, isovalerate production, and live weight.	[156]
AO fermentation extract (Amaferm^®^)	5 mg of AO/mL	Added to bacteria culture media	In vitro analysis	Ruminal bacteria interactions between antimicrobial compounds	Increased the growth rates of the fiber-digesting bacteria and the lactate-utilizing bacteria and diminished the negative effects of chlortetracycline or neomycin compounds.	[24]
AO fermentation extract (Amaferm^®^)	1.2 g/L	Incubated with ground fibrous feedstuffs with rumen fluid and buffer inoculum	In vitro analysis	Fiber degradation	Increased NDF and ADF degradations.	[157]
AO extract with yeast culture	90 g/d of AO and yeast culture	Supplemented into the basal diet	4 non-lactating Holstein cows	Nutrient digestibility, ruminal fermentation parameters	Increased digestibility of CP, hemicellulose and DM increased acetate to propionate ratio.	[158]
AO fermentation extract (Amaferm^®^)	5 g/day	Supplemented into the basal diet	64 multiparous Chinese Holstein cows	Rumen microbial community and activity	Increased populations of rumen fungi, increased MCP and the activity of carboxymethylcellulase (CMCase).	[159]
AO extract with yeast culture	6.0 and 26 g/kg of DM	Supplemented into a pelleted calf starter	40 buffalo calves	Nutrient digestibility	Increased total tract digestibility, higher average daily gain, higher digestibility of fiber.	[160]
AO culture	100 μL/50 mL incubation medium	Added to the cultures of ruminal microorganisms	In vitro	Ruminal fermentation	Decreased propionate, butyrate, and total volatile fatty acids. Increased the acetate:propionate ratio.	[161]
AO fermentation extract (Amaferm^®^)	1.5 g/d	Supplemented into the TMR	64 lactating cows	Digestibility of CP, NDF, and DM	Increased DMI.	[162]
AO culture	900 mg/kg	Supplemented into the ration including corn, hay, steam bone meal, and wheat bran	8 crossbreds wethers	Fiber digestion	Increased ADF degradation.	[163]
AO fermentation extract non-ionic surfactant	100 g/d (prepartum) 150 g/d (postpartum)	Supplemented into the TMR	40 Holstein dairy cows	Dry matter intake	Greater DMI in the transitional period, Increased milk fat content.	[164]
AO fermentation extract	2 g/d	Top-dressed on texturized starter ration	52 bull calves	Growth rate at weaning age and rumen development	Tended to increase ADG.	[165]
AO culture extract	0.25 mg/mL	Added to the fermentation of a basal ration	In vitro (Rusitec)	Stoichiometry of the rumen fermentation, microbial community	Eliminated the transient fall in pH, increased the acetate:propionate ratio and butyrate proportion, increased ammonia concentration, doubled the number of total viable bacteria, reduced protozoal numbers.	[76]
AO culture	3 g/d	Supplemented into the diet (70% concentrate and 30% forage)	2 dry and 2 lactating cows	Nutrient utilization	Increased rumen and total tract digestibility of fiber fractions.	[77]
AO fermentation extract	3 g/d	Top-dressing at the morning feeding plus 87 g of ground sorghum	47 lactating cows (20 primiparous and 26 multiparous)	Feed digestibility	Increased digestibility of DM, CP, NDF, and ADF.	[166]
AO fermentation extract	0.6 g/d	Supplemented into the TMR	4 Holstein dairy cows	Rumen operation condition	Increased pH, decreased the changing time.	[167]
AO fermentation extract	27 g/d	Added to a supplement based on soybean meal	6 non-lactating beef cows	Ruminal fermentation	Higher total VFA, pH tended to be lower.	[168]

Abbreviations: *Saccharomyces cerevisiae* (SC), *Aspergillus oryzae* (AO), *Aspergillus niger* (AN), dry matter (DM), crude protein (CP), neutral detergent fiber (NDF), acid detergent fiber (ADF), average daily gain (ADG), total mixed ration (TMR), fungal amylase unit (FAU), dry matter intake (DMI).

## Data Availability

Data cited in this review manuscript were collected from published literature presented in the list of references.
